# Weil's Disease: A Rare Cause of Jaundice

**DOI:** 10.7759/cureus.8428

**Published:** 2020-06-03

**Authors:** Jinendra Satiya, Niyati M Gupta, Malav P Parikh

**Affiliations:** 1 Internal Medicine, Metropolitan Hospital, New York City, USA; 2 Gastroenterology and Hepatology, State University of New York Downstate Medical Center, Brooklyn, USA

**Keywords:** jaundice, leptospirosis, liver function

## Abstract

Weil’s disease is a complication of untreated leptospirosis and can be fatal. Awareness of leptospirosis and its hepatic manifestations are limited. We report the case of a 50-year-old man with gastrointestinal symptoms and a cholestatic pattern of liver injury diagnosed with Weil’s disease. The patient showed remarkable improvement after treatment with appropriate antibiotics with normalization of liver function in one month. This case highlights the importance of recognizing leptospirosis and its myriad presentations.

## Introduction

Leptospirosis is a bacterial zoonosis seen globally, with an estimated annual case burden of 853,000 with 48,000 deaths [1]. The clinical presentation can be non-specific and easily confused with other viral diseases, such as dengue. Left untreated, leptospirosis can progress to life-threatening complications, including renal failure, respiratory failure and shock. Given its presentation with jaundice and the potential to cause acute liver failure, there exists a need to raise awareness of the disease amongst gastroenterologists. We report a case of leptospirosis presenting with gastrointestinal symptoms and abnormalities in liver function tests.

## Case presentation

A 50-year-old male with no significant past medical history presented to the hospital with nausea, vomiting, jaundice and right upper quadrant (RUQ) abdominal pain since one week. He was afebrile and his physical exam was remarkable for scleral icterus, but negative for hepatosplenomegaly or Murphy's sign. He denied recent antibiotic use, but reported a history of direct exposure to sewage water for the past one week. Routine laboratory tests showed thrombocytopenia (platelets: 48,000/uL), with normal hemoglobin and white blood cell counts. Routine chemistry showed acute kidney injury (AKI) with a creatinine of 4.39 mg/dl and a predominantly cholestatic pattern of liver injury, with a markedly elevated total bilirubin of 17.6 mg/dl (conjugated: 11.9 mg/dl), normal alkaline phosphatase, mild elevation of liver enzymes (alanine aminotransferase [ALT]: 77 U/L; aspartate aminotransferase [AST]: 124 U/L) and a normal international normalized ratio (INR) (Table 1).

**Table 1:** Abnormal laboratory tests AST, aspartate aminotransferase; ALT, alanine aminotransferase; INR, international normalized ratio

Laboratory test	Value
AST	124 U/L
ALT	77 U/L
INR	1.0
Total bilirubin	17.6 mg/dl
Direct bilirubin	11.9 mg/dl
Platelets	48000 /uL
Creatinine	4.39 mg/dl

Hepatitis panel, anti-mitochondrial antibody, IgG4 levels, HIV, haptoglobin and peripheral blood smear for schistocytes, acetaminophen level, serum alcohol level and urine drug screen were negative.

Ultrasound of the RUQ abdominal pain showed biliary sludge and non-specific gallbladder (GB) wall thickening, but no cholelithiasis or biliary duct dilatation. Magnetic resonance cholangiopancreatography (MRCP) showed similar findings and no choledocholithiasis. A hepatobiliary iminodiacetic acid (HIDA) scan revealed non-visualization of GB, consistent with a failure of the liver to excrete radioisotope into biliary tree and intense intrahepatic cholestasis. The total bilirubin peaked to 19.5 mg/dl and a liver biopsy was performed, which revealed apoptotic hepatocytes, canalicular cholestasis and non-specific patchy lobular inflammation, but no steatosis, ballooning, mallory hyaline bodies or fibrosis.

Given exposure to sewage water, AKI and hepatic involvement, leptospirosis serologies were sent. Leptospirosis IgM antibodies were positive on dot blot assay and a diagnosis of Weil’s disease was confirmed. The patient was started on doxycycline, intravenous fluids and close monitoring of the laboratory parameters, and showed significant improvement in bilirubin levels. AKI and thrombocytopenia resolved. Liver function test normalized completely on follow-up at one month.

## Discussion

Leptospirosis can present a significant diagnostic challenge, especially in tropical and subtropical areas. Rodents are the main animal reservoirs. Transmission most commonly occurs from animal urine and through contact with freshwater bodies. Human-to-human transmission is rare. The incubation period is from 2 to 21 days with sudden onset of symptoms. The primary phase constitutes the initial symptoms of fever, headache and myalgias. Abdominal pain, conjunctival suffusion and a skin rash may also be reported. Jaundice is seen in severe cases.

The second “immune” phase is characterized by a fever spike and coincides with the development of IgM antibodies. Weil’s disease is a triad of hemorrhage, jaundice and renal failure, and is seen in less than one-third of cases [2]. Pulmonary alveoli and intra-cerebral are the most common sites of hemorrhage. Only a very small proportion of infections lead to clinically significant disease, as few as one in 191 infections [3]. It is commonly seen in sewage workers, farmers and hunters and is associated with outdoor activities such as kayaking, rafting and tramping [4]. Differential diagnosis includes dengue, typhoid, influenzae, human immunodeficiency virus seroconversion and other causes of fever of unknown origin.

Liver injury is common in leptospirosis. Typically, a mild elevation of liver enzymes is seen in the immune stage. Serum bilirubin and alkaline phosphatase may be elevated, constituting Weil’s disease. The presence of jaundice portends a poor prognosis with a reported mortality of 19.1% [5]. Acalculous cholecystitis and pancreatitis have also been reported as presenting manifestations of the disease [6]. Shintaku et al. reported a case of fulminant hepatic failure from leptospirosis [7]. At autopsy, the liver had the gross appearance similar to that seen in acute yellow liver atrophy. On microscopic evaluation, severe damage was seen in zone 3 (centrilobular region) with evidence of submassive necrosis of hepatocytes and remarkable hemorrhage. Complete disappearance of CD-31-positive, sinusoidal endothelial cells confirmed endothelial damage in zone 3 as the direct cause of fulminant hepatic failure, while zones 1 and 2 remained unaffected.

Lebreton et al. reported two cases of liver transplantation as a result of fulminant hepatic failure from leptospirosis [8]. Both cases had severe acute hepatitis with AST levels elevated to 16,650 and 17,000 IU/L, and ALT values of 7,900 and 9,500 IU/L. The maximum bilirubin value noted was 65.9 mg/dL with an INR of 6.76. Encephalopathy was not reported in either of these cases. Indocyanine green plasma disappearance rate (ICG-PDR) is a bedside test that can be used to assess dynamic liver function [9]. Kunikowska et al. first demonstrated its use in identifying deteriorating liver function in the setting of normal standard laboratory tests. They reported improving ICG-PDR levels despite a persistently elevated serum bilirubin, thereby demonstrating that serum bilirubin was an inaccurate marker of liver dysfunction in Weil’s disease and did not accurately reflect hepatic recovery in icteric leptospirosis [10]. ICG-PDR is an easy-to-use, quick and non-invasive tool that can be utilized to predict ICU admission in resource-limited endemic regions.

Early antibiotic therapy with supportive treatment and rehydration is recommended by the World Health Organization, and has been shown to improve outcomes if administered before the fifth day of disease. Effective antibiotic choices include penicillin, amoxicillin, doxycycline, third-generation cephalosporin and quinolones [11]. 

## Conclusions

Liver involvement in leptospirosis is an important determining factor for severity. Worse outcomes have been seen, especially if combined with acute renal failure. Serum bilirubin is not an accurate marker of liver function in these cases. ICG-PDR is an effective, non-invasive alternative that can be employed to estimate liver function and help in making key clinical decisions. A timely workup and early initiation of antibiotics are the mainstays of management. Recognition of a diagnosis of acute liver failure, despite the absence of encephalopathy in these cases, with appropriate referrals to transplant centers may be life-saving.
